# Analysis of Patulin in Apple Products Marketed in Belgium: Intra-Laboratory Validation Study and Occurrence

**DOI:** 10.3390/toxins15060368

**Published:** 2023-05-30

**Authors:** Emmanuel K. Tangni, Julien Masquelier, Els Van Hoeck

**Affiliations:** Sciensano, Chemical and Physical Health Risks, Organic Contaminants and Additives, Toxins Unit, Leuvensesteenweg 17, 3080 Tervuren, Belgium; julien.masquelier@sciensano.be (J.M.); els.vanhoeck@sciensano.be (E.V.H.)

**Keywords:** mycotoxins, patulin, apple, juices, puree, Belgium

## Abstract

Apple and apple derivatives (e.g., juices, puree) are the most important foodstuffs contaminated with patulin (PAT) in the human diet. To routinely monitor these foodstuffs and ensure that the PAT levels are below the maximum permitted levels, a method using liquid chromatography combined with tandem mass spectrometry (LC-MS/MS) has been developed. Afterwards, the method was successfully validated, reaching quantification limits of 1.2 μg/L for apple juice and cider, and 2.1 μg/kg for puree. Recovery experiments were performed with samples fortified with PAT in the range of 25–75 μg/L for juice/cider and 25–75 μg/kg for puree. The results show overall average recovery rates of 85% (RSD_r_ = 13.1%) and 86% (RSD_r_ = 2.6%) with maximum extended uncertainty (U_max_, k = 2) of 34 and 35% for apple juice/cider and puree, respectively. Next, the validated method was applied to 103 juices, 42 purees and 10 ciders purchased on the Belgian market in 2021. PAT was not found in the cider samples, but it was present in 54.4% of the tested apple juices (up to 191.1 μg/L) and 7.1% of the puree samples (up to 35.9 μg/kg). When comparing the results to the maximum levels set by Regulation EC n° 1881/2006 (i.e., 50 μg/L for juices and 25 μg/kg for puree for adults, and 10 μg/kg for infants and young children), exceedances were observed in five apple juices and one puree sample, for infants and young children. Using these data, a potential risk assessment for consumers can be suggested, and it is found that the quality of apple juices and purees sold in Belgium needs further regular surveillance.

## 1. Introduction

Children and adults favor apple puree, juice, nectar and compote due to their taste and nutritional aspects [1]. Moreover, juices with 100% fruit have gained popularity worldwide. However, apples can potentially be contaminated with patulin (PAT), produced by molds belonging to several genera, including *Penicillium*, *Aspergillus*, *Byssochlamys* and *Paecilomyces* [2]. This mycotoxin, mainly present in pome fruits like apples and pears, can also be found in blueberries, cherries, raspberries, strawberries, mangos and oranges [3]. In Belgium, apple products are the most consumed fruit, and the available occurrence data on apple products date from more than a decade ago [2]. The most common fungi found in decaying apples was *Penicillium expansum* [4], leading to worrying PAT levels recorded worldwide in foods [2] despite the existence of a multitude of PAT detoxification methods, such as washing, milling, physical removal of fungi and infected tissues, and thermal and non-thermal processing techniques. The use of non-mycotoxigenic fungi as a biocontrol agent showed also the ability to decrease the levels of PAT in foods [5,6,7,8,9,10,11,12,13,14]. Exposure to PAT exacerbated acutely toxic, genotoxic, cytotoxic, teratogenic and immunosuppressive effects. Mutagenic and gastrointestinal problems in rodents have been reported [15,16], whereas scarce data on the long-term toxic effects of PAT are available [17]. Based on all these properties, the International Agency for Research on Cancer classified PAT in group 3, i.e., not classifiable in terms of carcinogenicity for humans [18]. As a result, the maximum permitted levels (MLs) set by the European Commission were 50 μg/L in apple juices, 25 μg/kg in apple puree and 10 μg/kg in apple products for infants and young children [19,20]. Previous studies showed that apple juice and nectar are the main contributors to PAT intake [21,22]. In Belgium, apple juices were found to be PAT-contaminated up to 38.8 μg/L [23] and up to 123 μg/L [24]. Targeting the handicraft-made samples, Gillard et al. [25] confirmed that 18 samples screened out of 32 contained PAT above the European Regulatory limit of 50 μg/L. Reviewing PAT occurrence data, Vidal et al. [2] found that consumer’s risk assessment in Belgium was based on data collected in 2007, and concluded that it is necessary to update PAT exposure in countries where high PAT concentrations may be found in foods. More recent data on PAT occurrence profile in apple juices (cloudy and clear) and baby food based on apples are needed, and therefore, a reliable LC/MS-MS method was developed and validated for PAT analyses. Afterwards, this method was applied to apple juices, ciders and puree samples from the Belgian market.

## 2. Results and Discussion

### 2.1. Method Validation

The method was validated in-house, according to the guidance document EC DG SANTE 12089/2016 [26], the criteria given in Regulation (EC) No 401/2006 [27] and EC N° 2021/808 [28].

#### 2.1.1. Specificity and Carryover

No interferences were found in the analysis of blank extracts of non-contaminated apple juice, cider or apple puree. Regarding the tolerance of ±2.5% variation, the recorded retention times were satisfactory when running LC-MS/MS analyses of all targeted matrices. Moreover, the fragmentation of the PAT precursor ion 153.0 [M-H]^−^ gave two specific ion products (m/z 109.0/81.2) with a satisfactory variation < 20% [26]. Signal suppression has been appraised, and showed significant day-to-day variation (up to 60.5%) upon injection of the extracts of apple juice or apple puree (Table 1).

A stable isotopic-labeled internal standard (^13^C-PAT) was used to compensate for matrix effects. For carryover checking, no signal was detected for PAT upon the injection of blank extract after the highest calibration point. Otherwise, peak areas were often less than 1% of the areas of the first calibration concentration (0.625 μg/mL).

It is noticeable that no clean-up step using immuno-affinity or solid phase extraction was required, in contrast to the existing analytical methods, which are often based on the use of ethyl acetate extraction and enzymatic pre-treatment by pectinase [29]. These previous analytical methods were based on a reversed-phase HPLC combined with a photodiode array detector or an ultraviolet detector able to simultaneously quantify PAT and hydroxymethyl furfural (HMF, another compound present in apple juice) [9]. In contrast to these methods, the present LC-MS/MS method successfully avoids the HMF quantification, but involves the use of toluene, which is considered a burden to the environment and health.

#### 2.1.2. Linearity, Detection and Quantification Limits, Precisions

Coefficients of determination were always above 0.99 during the analyses, showing satisfactory goodness-of-fit (<20%) and good linearity for all calibration points [26].

For apple juice, the LOQ and LOD were 1.2 μg/L and 0.4 μg/L, whereas, for apple puree, these values was 2.1 μg/kg and 0.6 μg/kg, showing high sensitivity in the present method.

Table 2 presents the recoveries and precisions for different fortification levels.

The overall recoveries were 84.6% for apple juice and 85.7% for apple puree. Intra-laboratory repeatability (RSD_r_) percentages ranged between 8.6% and 13.1% for apple juice and 11.8% and 12.6% for apple puree.

Recovery and precision tests were in accordance with the requested criteria [27,28], indicating the good repeatability of the analytical method. The overall MU_(k=2)_ values were 34% and 35% for apple juice and apple, respectively.

#### 2.1.3. Accuracy Checking

Table 3 illustrates the accuracy of the present method, checked via participation in the proficiency test rounds organized by FAPAS.

Absolute Z scores ≤ 2.0 were always obtained, illustrating good performances as well as the reliability of the present LC-MS/MS method in PAT quantification, even in cloudy apple juices. It is worth noticing that in normal circumstances, about 95% of Z scores ranged −2 ≤ z ≤ 2, whilst occasional scores in the range of 2 < |z| < 3 can be observed at a rate of 1 out of 20, and Z scores > 3 can be found at a rate of about 1 out of 300 [30,31,32,33].

### 2.2. Real Sample Analysis

#### 2.2.1. PAT Contamination in Apple Juices

Table 4 presents the results of PAT incidence and concentrations according to their provenance, mode of production, processing and appearance.

PAT incidence levels in domestic (n = 63) and imported (n = 40) apple juices were 52.4 and 57.5%, with the respective mean concentrations of 10.3 ± 28.6 μg/L and 5.6 ± 15.6 μg/L. Considering the organic route of the raw materials used, incidences of positive samples were 52.7% for conventional samples (mean 7.9 ± 25.0 μg/kg) against 58.6% for organic apple juices (mean = 9.8 ± 23.1 μg/kg). However, similar incidences of PAT contaminations (57.1% versus 50.0%) were observed with no significant variation between PAT levels in cloudy samples (11.6 ± 29.7 μg/kg) against PAT contents in clear juices (3.5 ± 10.5 μg/kg), even when they were handicraft-made (17.8 μg/L versus 2.4 μg/L).

It is worth noticing that PAT concentrations surpassed the maximum permitted limit of 50 μg/L set by the European Union [20] in five apple juices, and reached 191.1 μg/kg in one domestic cloudy handicraft apple juice. One could conclude that occasional high batches of PAT contamination occurred in apple juices sold on the Belgian markets. The samples exceeding the legal limits were notified to the Belgian Federal Agency for the Safety of Food Chain (FASFC) in 2021, and the batches in question were withdrawn from the market in Belgium.

According to the previous studies performed in Belgium, the incidence of non-compliant samples was 79% (n = 29) for domestic apple juices and 86% (n = 14) for imported apple juices, with the highest level at 38.8 μg/L [23]. Higher contamination levels (up to 123.μg/L) were reported in 22 of the 177 apple juices examined [24]. PAT concentrations above the legal limit of 50 μg/L were observed in 18 out of 32 handicraft juices [25]. In the present study, the highest level was 191.1 μg/L, while in other European countries, high PAT contaminations were also observed in apple juices sold in the markets, namely, in Denmark (up to 122.5 μg/kg [34]), in Spain (up to 170 μg/kg [35]) and in Italy (up to 1.150 μg/L [36]). PAT does not accumulate in the body, but a high intake of this mycotoxin can cause gastrointestinal problems in humans [2]. Special attention should be given to children and infant exposure, as they constitute the most sensitive group at risk.

More recently, in Tunisia, an alarmingly high PAT contamination was found in 50% of the 214 tested samples, at levels ranging from 2 to 889 μg/L (average = 89 μg/L); 22% of the samples exceeded the EU regulatory limits, and the authors concluded that consuming fruit products marketed in Tunisia might be a health concern in this country. Concomitantly, they recommended the hard and fast surveillance of this toxin in fruit products in Tunisia [37]. The necessity of raising public awareness regarding the presence of PAT in foodstuffs should also be addressed both for the food processing sector, especially handicraft producers and home-growers, as well as for fruit consumers, in order to reduce the consumers’ exposure to this toxin.

#### 2.2.2. PAT Contamination in Cider

PAT was not found in the ten tested ciders from the Belgian market (concentration < 0.4 μg/L). In contrast to the previous study in Belgium, two ciders out of seven samples contained PAT, with the highest level at 6.1 μg/L [23]. The results of the present study corroborate well with the conclusion that PAT loads are much less prevalent in alcoholic apple beverages, since PAT concentrations may be decreased by the fermentation process along the food processing chain [38]. Despite non-detection and the low contamination in batches of cider samples, it is suggested that surveillance of this toxin in cider should be carried out in future studies with more samples, in order to draw more meaningful conclusions.

#### 2.2.3. PAT Contamination in Apple Puree

Forty-two apple purees were analyzed, including 16 domestic and 26 imported puree samples. They were made of organic starting materials (n = 18) or conventionally produced puree (n = 24). PAT was detected in only three apple puree, samples with an overall mean of 2.4 ± 5.6 μg/kg (Table 5).

The highest PAT concentration was 35.9 μg/kg, which exceeded the ML of 25 μg/kg set by Regulation (EU) n°1881/2006 [20]. This sample was industrially made of organic starting material.

It should be noted that “masked PAT” can be present in the juice, since PAT can be bound to the solid parts of the apple in the juice. Although “masked PAT” is not toxic on its own, it might be released into its toxic form in the digestive system of mammals. Therefore, the detected levels of PAT in food samples might be more important than its actual concentration [39]. Furthermore, PAT and its biomarkers are not usually included in the analytical methods for multi-mycotoxin determination, and therefore are not often targeted in multi-mycotoxin risk exposure [2]. Hence, focus should be placed on targeting PAT and its biomarkers in the development and validation of multi-mycotoxin methods using appropriate liquid chromatographic technologies. This may allow for refining PAT risk exposure amongst consumer’s in a multi-mycotoxin frame.

## 3. Conclusions

The present analytical study demonstrated the applicability of LC-MS/MS for PAT determination in apple products (e.g., apple juices, ciders and apple purees). Good performances were achieved by this method, which can be used for the routine screening of various selected samples in retail markets in Belgium. Except for ciders, PAT was found in apple juices at concentrations that surpassed the European maximum admissible levels in five apple juices and one apple puree. This study allowed for updating the occurrence data on PAT contamination in Belgium. Therefore, using these data to infer potential risk for consumers is recommended. The possible co-contamination of PAT with other contaminants also merits further risk assessment. Fruit derivatives coming from pears, cherries, raspberries, strawberries, mangos and oranges should also be checked in Belgium for PAT contaminations by taking into account seasonal and yearly variations.

## 4. Materials and Methods

### 4.1. Chemicals

Methanol (MeOH, ULC-MS absolute), acetonitrile (ACN, ULC-MS), acetic acid glacial AR, ammonium acetate ULC/MS, dimethyl sulfoxide AR (DMSO) and toluene AR were supplied by Biosolve B.V. (Valkenswaard, The Netherlands). Sodium chloride and magnesium sulfate were purchased from Merck (Darmstadt, Germany). Water (H_2_O) was generated by a Milli-Q Gradient purification system (Millipore, Billerica, MA, USA). Certified PAT (100 μg/mL) standard and a standard of ^13^C-labeled PAT (^13^C-PAT, 25 μg/mL) in ACN were purchased from Biopure-Romer Labs (Tulln, Austria). The ^13^C-PAT diluted in 50% MeOH% at 500 ng/mL was used as an internal standard (IS). From the ^12^C-PAT (100 μg/mL), two working solutions at 5 μg/mL and 500 ng/mL were prepared in 50% MeOH and used for the calibration and fortification experiments. All stock solutions were stored at −20 °C until use. Reference materials from FAPAS (Fera Science Ltd. National Agri-Food Innovation Campus. Sand Hutton, York, UK) were used for quality control.

### 4.2. Samples

Apple juice (n = 103), apple puree (n = 42) and apple ciders (n = 10) were procured in retail markets in 2021 in Belgium. They were randomly selected from general supermarkets and local markets during the so-called “fête des pommes”, annually organized in some villages in Wallonia (e.g., Ceroux, Burdinne, Héron, La Hulpe, Soumagne, Stoumont, etc.) in September and October. They were kept in the refrigerator (<+5 °C) till further analysis.

### 4.3. Sample Preparation

Homogenized apple juice (5.00 ± 0.02 mL) or homogenated apple purees (5.00 ± 0.02 g) were transferred into a 50 mL-polypropylene tube. For the recovery experiment, three subsamples were then fortified with PAT (25 μL of ^12^C-PAT, 5 μg/mL) and other subsamples were analyzed without any fortification. Fortified subsamples were left for 30 min before extraction. The extraction of PAT was carried out by adding 10 mL (for juices) or 20 mL (for puree) of ACN:Toluene:HAc (24:75:1). The mixture was then shaken for 1 h using a horizontal Reax 2 overhead shaker (VWR, Haasrode, Belgium). Afterwards, a 6 g MgSO_4_/1.5 g NaCl mixture was added.

The tubes were vigorously agitated by hand for 30 s and shaken for five minutes with the Reax overhead shaker (VWR, Haasrode, Belgium). Next, the tubes were centrifuged to precipitate the most undissolved salts and complete the phase separation. The salt-induced phase separation resulted in an upper ACN phase with the targeted analyte. An aliquot of 1 mL of the organic layer was taken, and 100 μL of DMSO was added. The ACN was evaporated, and 900 μL of salted solution (H_2_O:HAc, 99:1), mixed with ten g/100 mL of NaCl, was then added to the DMSO extract. The resulting solution was transferred into an Eppendorf tube, vortexed and centrifuged for 10 min at 13,000 rpm. Then, 95 μL of this solution was mixed with 5 μL of the IS (^13^C-PAT, 500 ng/mL), and then vortexed and injected (5 μL) into the LC-MS/MS system.

Material decontamination to prevent cross-contamination and specific safety handling of the mycotoxin solutions were applied as prescribed for good laboratory practice.

### 4.4. LC-MS/MS Analyses

The LC-MS/MS analyses were performed using a Xevo TQ-S mass spectrometer (Waters, Milford, Manchester, UK) with an ESI source. Chromatographic separation (7.5 min) was achieved using an HSS T3 C18 column (2.1 mm × 100 mm; 1.8 μm) maintained at 40 °C. Two elution solvents made of 0.05% acetic acid and 5 mM ammonium acetate in H_2_O (Phase A) and in MeOH (Phase B) were used. The 7.5 min run consisted of a linear increase in B from 1% to 20% over 5 min, with an instant increase to 100% B within 1 min at the flow rate of 0.5 mL/min. The initial conditions (99% A, 1% B) were restored for 1.5 min to re-equilibrate the column for the next run. A volume of 5 μL was injected, and the MS was operated in negative mode with a desolvation temperature of 450 °C, a desolvation gas flow of 1000 L/h, and a cone gas flow of 150 L/h. For MRM, the collision gas was argon on a pressure regulator (max gradient < 15,000 PSI). Ionization and MS/MS collision energy settings were optimized. The Masslynx and Targetlynx software allowed data acquisition and processing.

### 4.5. Method Validation

Validation parameters were estimated, such as the limit of detection (LOD), the limit of quantification (LOQ), recovery, repeatability, reproducibility and measurement uncertainty.

The linearity was checked in neat solvent and extracts of apple juice and apple puree samples fortified with PAT at concentrations ranging from 6.25 to 100.0 μg/kg. The goodness-of-fit was found to be satisfactory when the relative deviation was <20% [26]. The PAT-free apple juice and puree were used as “blank samples” for method validation. As described by Sulyok et al. [40], the matrix effects were calculated according to Equation (1). The matrix effect is considered tolerable when the extent of the signal suppression/enhancement is ≤20% [41].

(1) $$\mathrm{Matrix}\;\mathrm{effect}\;\left(\%\right)=\frac{{\mathrm{slope}}_{\mathrm{solvent}}-{\mathrm{slope}}_{\mathrm{matrix}}}{{\mathrm{slope}}_{\mathrm{solvent}}}\times 100$$

The limits of quantification (LOQs) were estimated using a theoretical signal-to-noise (S/N) of 10, whilst the limits of detection (LODs) were calculated with a signal-to-noise (S/N) of 3.

Based on the ML of 50 μg/L, recoveries and precisions were evaluated using samples fortified at 0.5xML, 1.0xML and 1.5xML in triplicate measurements for three days. Recovery (%) is the ratio between the measured and spiked concentration. Successful recovery rates are those between 70 and 105% [42] without any clean-up step, as recently performed by Shinde et al. [43], using the dispersive solid-phase extraction on primary and secondary amines or the HLB cartridge chosen by Seo et al. [44] as optimum SPE conditions.

Residual standard deviation RSD_r_ and RSD_wr_ values ≤ 20% were used to evaluate the repeatability (intraday precision) and the intermediate precision (inter-day precision) [28].

Extended measurement uncertainty MU_(k=2)_ was done by multiplying the standard deviation by a coverage factor (k = 2) with 95% confidence. The precisions were determined according to the performance criteria: MU_(k=2)_ < MUmax_(k = 2)_ = 0.66 x RSD_R_ (calculated using Horwitz Equation (2)).

RSD_R_ = 2^[1−0.5logC]^ (2)

Participation in the proficiency testing (PT) rounds organized by FAPAS allowed us to evaluate the trueness. To demonstrate good performance, Z scores should be between −2 and +2 [30,31,32,33].

### 4.6. Quality Assurance and Quality Control

Three different types of quality controls were used.

*Selectivity and carryover checking*: The absence of detectable peaks of PAT in a blank extraction solvent injected directly after the highest calibration point was checked. For retention time stability, a maximum of 2.5% difference in the retention times should be established between the solvent-based standards and fortified samples. Note that each series of analyses included a solvent-based control with a concentration of 50 ng/mL, injected at least three times to verify that the variation should not be higher than 10% between two consecutive injections and not more than 20% over the entire run. This is repeated every ten injections and at the end of each series.

*Recovery checking*: In each analytical series, a blank sample and one quality control sample (i.e., sample fortified with PAT at 5 μg/mL before extraction) were analyzed following the same protocol as the unknown samples. The recovery was defined as the proportion of the amount of analyte added versus the amount detected after correction for the matrix effect. For PAT, the recovery should be between 70% and 105% [42].

*Trueness checking*: The incurred reference material was systematically included in each series of analyses to build a control chart intended to flag any deviation or trend concerning the acceptable recovery rates in the reference material.

Finally, the analysis was ISO 17025-accredited by the Belgian accreditation board BELAC, and can thus be recommended for regulatory testing purposes by food safety authorities.

## Figures and Tables

**Table 1:** Variation in LC-MS/MS signal suppression of apple juice and puree extracts.

Extracts	Day 1	Day 2	Day 3
Apple juice	20.1 ± 8.8%	60.5 ± 5.8%	23.3 ± 5.4%
Apple puree	21.9 ±10.7%	16.1 ± 5.7%	9.8 ± 6.7%

**Table 2:** Recovery, repeatability, intra-laboratory reproducibility and measurement uncertainties.

Matrices	Nominal PAT Concentrations	Number of Replicate	Measured PAT Concentrations	Recovery	Intra Laboratory Repeatability u_(k = 1)_		Extended Measurement Uncertainty MU_(k = 2)_	
	μg/kg	n *	μg/kg	%	μg/kg	%	μg/kg	%
Apple juice	25	9	24.24	97.0	3.17	13.1	6.35	26.2
	50	9	46.24	92.5	4.28	9.3	8.55	18.5
	75	9	66.53	88.7	5.56	8.4	11.11	16.7
Apple puree	25	9	23.26	93.0	2.92	12.6	5.84	25.1
	50	9	43.24	85.5	5.11	11.8	10.22	23.6
	75	9	66.11	88.1	7.92	12.0	15.85	24.0

*: 3 replicates/day × 3 days.

**Table 3:** Analytical performances via FAPAS proficiency test rounds.

FAPAS PT Rounds						
Years	Matrices	Assigned Value	Standard Deviation for Proficiency	Obtained Results	Z Scores	Reference Test Report FAPAS
2020	Apple juice (cloudy)	18.6 μg/L	4.10 μg/L	21.3 μg/L	0.7	1674 [30]
2021	Apple puree	14.6 μg/kg	3.21 μg/kg	14.3 μg/kg	−0.1	1676 [31]
2021	Apple juice (cloudy)	18.2 μg/L	4.00 μg/L	11.8 μg/L	−1.6	1677 [32]
2022	Apple juice (cloudy)	14.4 μg/L	3.17 μg/L	17.1 μg/L	0.8	1680 [33]

**Table 4:** Occurrence of PAT in apple juices, taking into account their provenance, mode of production and cloudiness status.

	Positive/Total	%	Number of Samples			Mean * ± SD μg/L	Median	95th/99th	Maximum
			<10 μg/L	10–50 μg/L	>50 μg/L		μg/L	Percentiles μg/L	μg/L
Provenance									
Domestic	33/63	52.4	23	7	3	10.3 ± 28.6	1.7	41.3/130.8	191.1
Imported	23/40	57.5	21	0	2	5.6 ± 15.6	1.7	11.1/72.8	76.6
Mode of production									
Conventional	39/74	52.7	31	6	2	7.9 ± 25.0	1.3	25.6/120.1	191.1
Organic	17/29	58.6	13	1	3	9.8 ± 23.1	1.8	72.7/81.9	84.0
Appearance									
Cloudy	36/63	57.1	25	7	4	11.6 ± 29.7	2.6	73.2/130.8	191.1
Clear	20/40	50.0	19	0	1	3.5 ± 10.5	0.7	8.2/44.2	66.9
Processing and Appearance									
*Industrial*	*34/65*	*52.3*	*30*	*2*	*2*	*4.9 ± 12.8*	*1.3*	*16.7/70.4*	*76.6*
Cloudy	17/33	51.5	14	2	1	6.0 ± 13.9	1.2	21.5/60.4	76.6
Clear	17/32	53.1	16	0	1	3.8 ± 11.7	1.4	5.7/48.0	66.9
*Handicraft*	*22/38*	*58.9*	*14*	*5*	*3*	*14.5 ± 36.0*	*2.4*	*85.5/155.1*	*191.1*
Cloudy	19/30	63.3	11	5	3	17.8 ± 40.0	2.9	89.4/162.9	191.1
Clear	3/8	37.5	3	0	0	2.4 ± 3.8	0.2	8.6/8.8	8.8
Total juices	56/103	54.4	44	7	5	8.5 ± 24.4	1.7	41.3/93.6	191.1

*: Non-detected (PAT concentration < LOD), assumed to contain half of the LOD value. Trace levels (LOD < concentrations < LOQ) were assumed to contain LOQ values.

**Table 5:** PAT levels in apple purees according to the provenance, and the organic grade of their production and processing.

	Positive/Total	%	Number of Samples			Mean * ± SD μg/kg	Median	95th/99th	Max
			<10 μg/kg	10–25 μg/kg	> 25 μg/kg		μg/kg	Percentiles μg/kg	μg/kg
Provenance									
Domestic	1/16	6.3	1	0	0	0.5 ± 0.8	0.3	1.1/3.1	3.6
Imported	2/26	7.7	1	0	1	2.0 ± 7.1	0.3	6.7/29.1	35.9
Mode of production									
Conventional	1/24	4.2	1	0	0	0.4 ± 0.7	0.3	0.3/2.8	3.6
Organic	2/18	11.1	1	0	1	2.8 ± 8.5	0.3	12.9/31.3	35.9
Processing									
Industrial	3/41	7.3	2	0	1	1.5 ± 5.7	0.3	3.6/25.1	35.9
Handicraft	0/1	0	0	0	0	*-*	-	-	-
Total puree	3/42	7.1	2	0	1	2.4±5.6	0.3	3.4/24.8	35.9

*: Non-detected (PAT concentration < LOD), assumed to contain half of the LOD value. Trace levels (LOD < concentrations < LOQ) were assumed to contain LOQ values.

## Data Availability

Data are unavailable due to privacy.
